# On the Ligation of Arteries

**Published:** 1857-01

**Authors:** T. P. Gibbons


ART. III.—*On the Ligation of Arteries.* By T. P. GIBBONS, M.D.

THE danger of secondary hemorrhage is well known to be one of the great drawbacks to the ligation of the large and deep-seated arteries. This is particularly true with regard to the femoral artery, owing to the number of its collateral branches. Indeed, so many unfortunate results, from this cause, have occurred in the ligation of this vessel, that some surgeons prefer, rather than resort to it, in certain cases, to amputate the limb.

The method usually recommended for the performance of ligation, is to cut down upon the vessel and open the sheath, without disturbing the tissues more than just sufficient to carry the aneurismal needle, armed with the ligature, around it. By this plan of procedure, it is contended, that the danger of extensive sloughing is avoided. This is unquestionably true. But how is the danger consequent upon the application of the ligature immediately below a large collateral branch, to be obviated by such a process?

Of all the animal tissues, that of arteries is least liable to slough; therefore, taking this fact in consideration, it appears a fair inference, that by exposing the artery as much as may be necessary in the method for applying the ligature, presently to be described, the danger of secondary hemorrhage is, to a considerable extent, lessened.

Some two years ago, I witnessed the operation of ligature of the femoral artery, for an extensive osseous aneurism in the head of the fibula. The case progressed favorably, the ligature came away at the end of six weeks, and the wound closed. The operation, so far as the cure of the aneurism was concerned, was successful. The man subsequently died from an attack of delirium tremens, and an opportunity was afforded of examining the parts. The ligature had been applied midway between two collateral branches, about an inch and a quarter apart. An organized plug, upon each side of the ligature, had produced complete obliteration of the calibre of the vessel.

The peculiar plan of the operation was as follows. The artery was exposed in its sheath, to the extent of half an inch; a grooved director was carried *obliquely* under it, and raised so as to allow the ends of the instrument to rest upon the edges of the wound; the director was then brought round at right angles to the course of the vessel, and an eyed probe, armed with a ligature, carried along the groove. The artery was tied at this point.

The mechanism of the process is very simple. When the director is carried obliquely under the vessel, as the primary step, and subsequently moved round at right angles with it, it will be observed that the position which the instrument holds beneath the artery, is such as to insure the application of the ligature midway between the two nearest points of resistance; which points usually to the connection of the collateral branches. The reason for its occupying this particular position is sufficiently evident: in moving it from a branch the resistance becomes less, while in moving it towards one, the resistance is of course increased; the consequence is, that the director, when placed at right angles with, and under the vessel, naturally assumes a position where the two forces act equally, that is, equidistant from the two points of resistance.

That the danger of destroying the vitality of an artery, is not so great as is usually supposed, may be inferred from the following case. Some time ago, I had occasion to cut down upon, and tie the radial artery for secondary hemorrhage, which occurred from a wound at the wrist. The artery was exposed and separated from its sheath for the distance of an inch, and the ligature applied at the distal extremity of the wound. Everything progressed favorably, the ligature came away about the usual time, the wound healed kindly, and there was no cause to regret having isolated the vessel from its cellular attachments. We see the same indisposition to slough on the part of the arteries frequently manifested when they are exposed in deep ulcerating wounds, where the process of destruction has involved almost every structure in the neighborhood, including the cellular tissue immediately around them; and yet, under such adverse circumstances, their integrity is perfectly preserved. If then, nature has endowed these vessels with such remarkable powers of resistance, why should surgeons hesitate to act on the suggestion so plainly thrown out, and reap the obvious advantage which isolation of the artery, and separation from its vascular conduits, will afford them in the operation of ligation for aneurism?

